# Subcostal robotic-assisted pulmonary resection: Left lingular segmentectomy

**DOI:** 10.1016/j.xjtc.2022.08.018

**Published:** 2022-09-06

**Authors:** Rajkamal Vishnu, Woohyun Jung, Beatrice Chia-Hui Shih, Yoohwa Hwang, Jae Hyun Jeon, Sukki Cho, Kwhanmien Kim, Sanghoon Jheon

**Affiliations:** Division of Thoracic Surgery, Department of Thoracic and Cardiovascular Surgery, Seoul National University Bundang Hospital, Bundang-gu, Seongnam-si, Gyeonggi-do, South Korea

**Figure:**
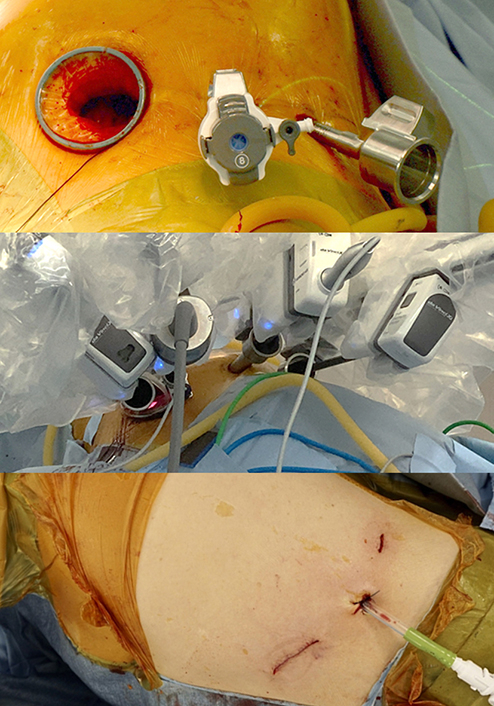
Subcostal robotic-assisted pulmonary resection.

**Central Message**

Subcostal robotic-assisted pulmonary resection is feasible for left lingular segmentectomy while preserving the intercostal spaces.

There is continued interest in finding an optimal approach for thoracic surgery. The intercostal space has been accepted as the standard route for video-assisted thoracic surgery (VATS) and robot-assisted thoracic surgery (RATS). However, the intercostal approach causes postoperative chronic pain and related complications due to intercostal nerve injury. To overcome the shortcomings of the intercostal approach, the subcostal VATS has been proposed.^1^ However, the long distance from the costal arch to the hilum and the interference from the hip and abdomen make this procedure difficult. Therefore, we introduced the subcostal RATS, which is less technically demanding than subcostal VATS while maintaining the advantages of the subcostal route.

## Technique

**Figure E1:**
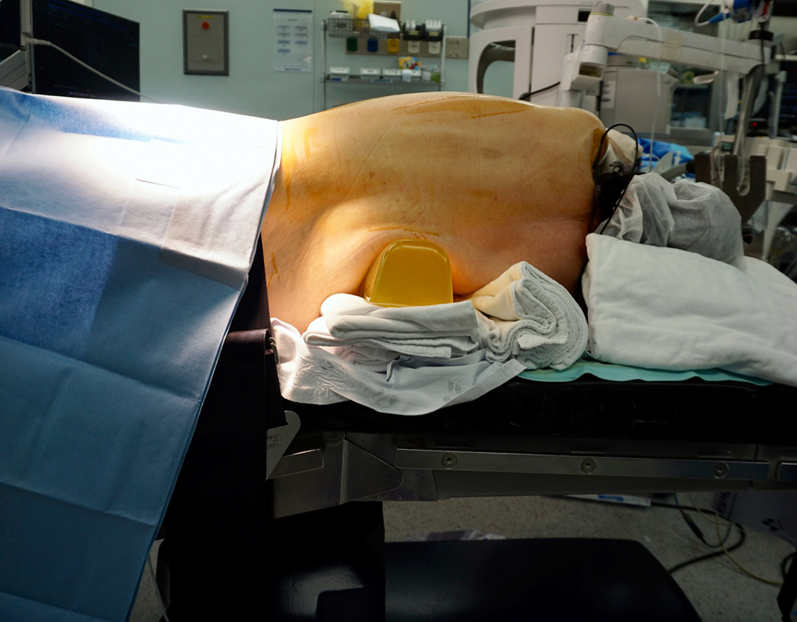
Patient position for subcostal robot-assisted thoracic surgery.

We performed the subcostal RATS left lingular segmentectomy for pulmonary arteriovenous malformation. The patient was intubated with a double-lumen endotracheal tube and placed in a right lateral decubitus position. We try to minimize the interference of the patient's hip and abdomen with the robotic arms by placing a pillow under the flank and arching the patient's back downward (Figure E1). The requirement for informed consent was waived because of the retrospective study design (institutional review board number and date of approval: B-2206-763-102, June 3, 2022).

**Figure 1:**
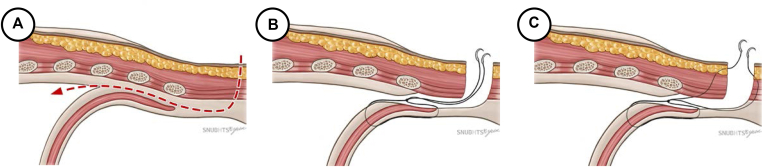
Subcostal incision. A, Anatomic scheme for subcostal working window incision; B, pre-emptive sutures for preventing diaphragm injury during surgery; and C, closure of the subcostal incision.

**Figure 2:**
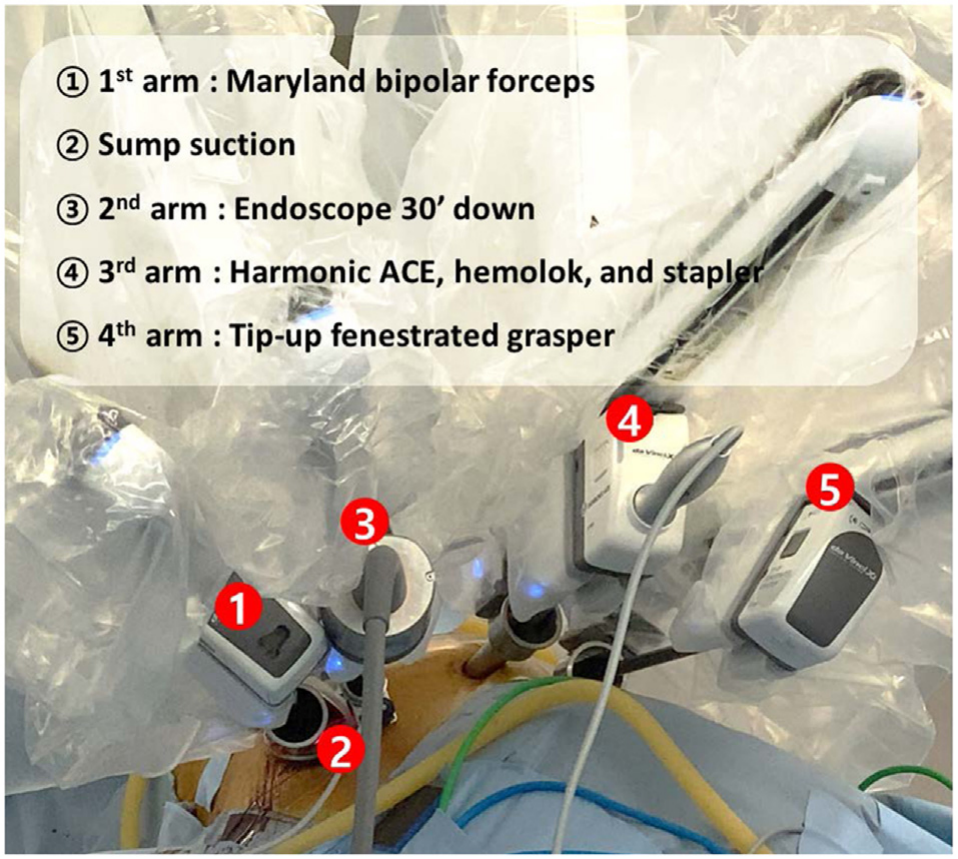
Port access and their use for subcostal robotic surgery for left lingular segmentectomy.

A 4-cm long working port was made where the subcostal arch and the midclavicular line meet. The subcutaneous tissue and oblique muscles were incised until the transverse abdominalis fascia was visible. The pleura was accessed by tunneling below the costal cartilages and above the diaphragm using mosquito forceps. To protect the diaphragm during surgery, we sutured the cut edge of the diaphragmatic parietal pleura to the transverse abdominalis fascia preemptively and applied a wound protector (Figure 1, *A* and *B*). Subcostal 12-mm and 8-mm ports were created. Each port was kept 4 cm apart. The first and second arms were docked to ports placed on either end of the working port. The sump suction was placed within the working port. The remaining arms were docked to the 12-mm and 8-mm ports, respectively (Figure 2). The lingular segmental artery and vein were divided. The segmental bronchus was cut open and later stapled. The intersegmental plane was divided under the guidance of Firefly (Intuitive) (Video 1). A 24-F chest tube was inserted through a 12-mm port. The proximal end of an oblique muscle, the cut edge of the diaphragmatic parietal pleura, and the distal end of an oblique muscle were sutured together, and the rest of the wound was closed (Figure 1, *C*).

## Comment

The VATS has revolutionized the management of lung cancer treatment.^2^ The intercostal VATS has been refined over decades and established as the standard for thoracic surgery. RATS has been introduced for thoracic surgery recently, but it shares the same problem caused by intercostal space usage.

Subcostal VATS has recently been proposed to overcome the limitation of intercostal usage.^1^ However, the subcostal VATS is challenging to perform and unsafe for the following reasons: Most VATS instruments are rigid and unable to be angulated, and the distance from the subcostal arch to the hilum is far; therefore, range of the instrument's motion is limited. The patient's abdomen and hip interfere with handling the instruments. As a result, the expertise is required to conduct subcostal VATS.

To maintain the benefits while overcoming the limits of subcostal VATS, we introduced subcostal RATS. Because RATS instruments are much longer, the hilum can be easily reached from the subcostal arch. This long distance makes it easier to control the robotic stapler. The robotic carriage adapter is oriented upside down, so the interferences from the hip and the abdomen are minimized. However, the subcostal RATS also shares the same obstacle as VATS; the camera is rigid and cannot provide a superior-to-inferior view, which makes it hard to dissect the structures above the remaining bronchus safely. Therefore, we adopted the inferior-to-superior approach and bronchotomy-and-closure maneuver.

The subcostal RATS is a novel access strategy with potential benefits to patients. It spares the intercostal nerves, which might provide a swift recovery. Unlike the intercostal approach, there are no bony structures below the subcostal incision. Therefore, handling the instrument upward would be easier without pressing down the rib, and a smaller incision is enough to retrieve the specimen. Robotic surgery reigns advantageous to VATS, as it provides enhanced visualization and expanded degrees of dexterity and surgeon autonomy.^3^ If future research provides evidence of the safety and advantages of the subcostal incision over the intercostal, the subcostal RATS could be adopted as a choice of treatment in a new era of minimally invasive surgery. In conclusion, we report the subcostal RATS for the first time.

## Supplementary Data

Video 1 Subcostal robot-assisted left lingular segmentectomy. Video available at: https://www.jtcvs.org/article/S2666-2507(22)00459-X/fulltext.
