# Supplementary figures and images for: Subcostal robotic-assisted pulmonary resection: Left lingular segmentectomy
Source: JTCVS Tech. 2022 Sep 6;16:149–52. doi: 10.1016/j.xjtc.2022.08.018 (PMC9735356; doi:10.1016/j.xjtc.2022.08.018)

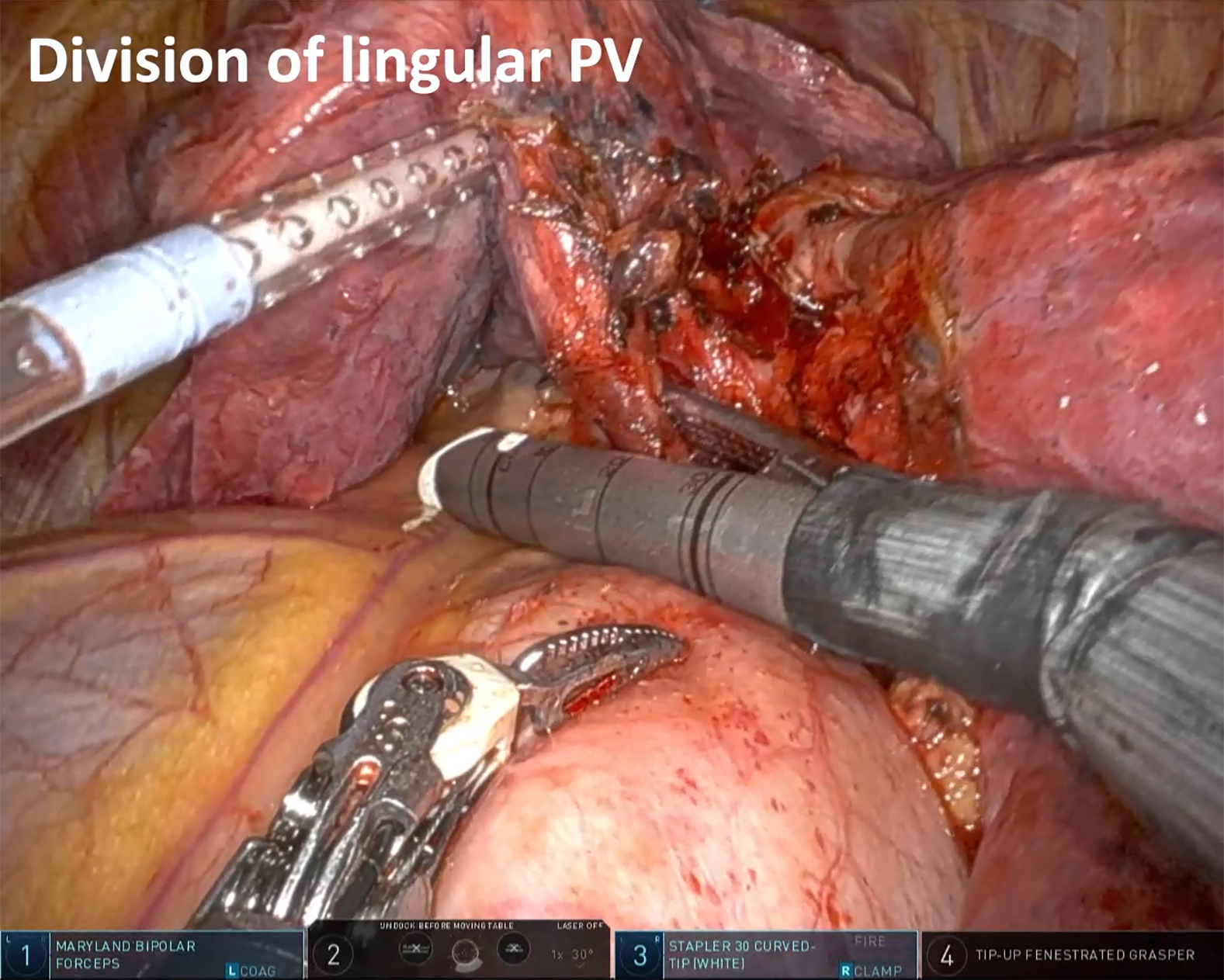

Supplement: Video 1 — Subcostal robot-assisted left lingular segmentectomy. Video available at: https://www.jtcvs.org/article/S2666-2507(22)00459-X/fulltext. [file fx2.jpg]
